# Effect of Propionic Acid on Diabetes-Induced Impairment of Unfolded Protein Response Signaling and Astrocyte/Microglia Crosstalk in Rat Ventromedial Nucleus of the Hypothalamus

**DOI:** 10.1155/2022/6404964

**Published:** 2022-01-22

**Authors:** Larysa V. Natrus, Yulia S. Osadchuk, Olha O. Lisakovska, Dmytro O. Labudzinskyi, Yulia G. Klys, Yuri B. Chaikovsky

**Affiliations:** ^1^Department of Modern Technologies of Medical Diagnostics and Treatment, Bogomolets National Medical University, Kyiv 03115, Ukraine; ^2^Department of Histology and Embryology, Bogomolets National Medical University, Kyiv 03115, Ukraine; ^3^Department of Biochemistry of Vitamins and Coenzymes, Palladin Institute of Biochemistry of the National Academy of Sciences of Ukraine, Kyiv 01054, Ukraine

## Abstract

**Background:**

The aim was to investigate the influence of propionic acid (PA) on the endoplasmic reticulum (ER), unfolded protein response (UPR) state, and astrocyte/microglia markers in rat ventromedial hypothalamus (VMH) after type 2 diabetes mellitus (T2DM).

**Methods:**

Male Wistar rats were divided: (1) control, (2) T2DM, and groups that received the following (14 days, orally): (3) metformin (60 mg/kg), (4) PA (60 mg/kg), and (5) PA+metformin. Western blotting, RT-PCR, transmission electron microscopy, and immunohistochemical staining were performed.

**Results:**

We found T2DM-associated enlargement of ER cisterns, while drug administration slightly improved VMH ultrastructural signs of damage. GRP78 level was 2.1-fold lower in T2DM vs. control. Metformin restored GRP78 to control, while PA increased it by 2.56-fold and metformin+PA—by 3.28-fold vs. T2DM. PERK was elevated by 3.61-fold in T2DM, after metformin—by 4.98-fold, PA—5.64-fold, and metformin+PA—3.01-fold vs. control. A 2.45-fold increase in ATF6 was observed in T2DM. Metformin decreased ATF6 content vs. T2DM. Interestingly, PA exerted a more pronounced lowering effect on ATF6, while combined treatment restored ATF6 to control. IRE1 increased in T2DM (2.4-fold), metformin (1.99-fold), and PA (1.45-fold) groups vs. control, while metformin+PA fully normalized its content. The Iba1 level was upregulated in T2DM (5.44-fold) and metformin groups (6.88-fold). Despite PA treatment leading to a further 8.9-fold Iba1 elevation, PA+metformin caused the Iba1 decline vs. metformin and PA treatment. GFAP level did not change in T2DM but rose in metformin and PA groups vs. control. PA+metformin administration diminished GFAP vs. PA. T2DM-induced changes were associated with dramatically decreased ZO-1 levels, while PA treatment increased it almost to control values.

**Conclusions:**

T2DM-induced UPR imbalance, activation of microglia, and impairments in cell integrity may trigger VMH dysfunction. Drug administration slightly improved ultrastructural changes in VMH, normalized UPR, and caused an astrocyte activation. PA and metformin exerted beneficial effects for counteracting diabetes-induced ER stress in VMH.

## 1. Introduction

Obesity, metabolic syndrome (MS), and type 2 diabetes mellitus (T2DM) are among the most serious health problems in the 21^st^ century. According to the data of the World Health Organization, more than 1 billion people around the world suffer from overweighting and/or obesity [1]. In recent years, it has been established a complex multifactorial interconnection between obesity, MS, and T2DM—three related conditions that are linked by a number of pathophysiological mechanisms. The state of insulin resistance, which is the common link between obesity and MS, leads to glucose metabolism disorders, dyslipidemia, high blood pressure, endothelial dysfunction, inflammatory dermatoses, and other diseases and is characterized by increased inflammatory cytokine activity [2, 3]. Over the last years, significant efforts have been made to discover cellular and molecular pathogenic mechanisms underlying MS. However, they turned out to be complex, still debated, and remain to be fully elucidated. Moreover, current preventive and/or therapeutic options for MS/T2DM include dietary control and regular exercises and are individually limited.

In addition to metabolic derangements in peripheral organs, there is a bidirectional link between obesity and perturbation of brain function; especially those brain areas that regulate energy homeostasis and systemic metabolism, e.g., *hypothalamus*. The *hypothalamus* controls food intake and body weight. Hypothalamic dysregulation may be one of the underlying mechanisms of abnormal glucose metabolism and T2DM and is associated with the development of low-grade inflammation and impaired neurogenesis [4]. Moreover, it is known that microglia and astrocytes are implicated in the pathophysiology of obesity and diabetes [5]; however, mechanisms of astrocyte/microglia crosstalk in the hypothalamus on the background of T2DM still remain undiscovered.

One of the causes underlying the disturbance of the central mechanisms maintaining homeostasis is impairments in the activity of neurons in the anterior hypothalamus. Using electrophysiological approach, we have previously analyzed the response of single neurons to thermo-, press-, osmo-, and glucostimulation and found that the only 19% of neurons are monosensory in the anterior hypothalamus. At the same time, the majority of neurons are polysensory and equally sensitive to fluctuations of combined visceral stimuli. Thus, the existence of polysensory neurons is mediated by the absence of an isolated change in the only one homeostatic parameter and that the fact that fluctuations of any constant of homeostasis definitely cause a shift in other homeostatic parameters [6]. However, this complex functional organization of neurons of the anterior hypothalamus is combined with homogeneity of the neurons forming the regulatory centers on the morphological level. Therefore, it may be suggested that all homeostatic shifts, including combined hyperlipidemia and hyperglycemia, are reflected in the hypothalamic neurons at the molecular level by influencing regulatory signaling pathways and networks.

Besides the cellular level, there are molecular derangements that may represent concurrent reasons and consequences of the state of stable hyperglycemia. Pathological stimuli including hyperglycemia deregulate normal endoplasmic reticulum (ER) functioning and unfolded protein response (UPR) signaling [7]. The particular importance of ER stress-responsive molecules in insulin biosynthesis, gluconeogenesis, insulin resistance, glucose intolerance, and their influence on hypothalamic functions should be highlighted. An understanding of the pathogenic mechanism of hypothalamic disturbances from the aspect of ER stress might be beneficial for developing new therapeutic strategies.

Therefore, it is of great significance to study the link between hypothalamus dysfunction and pathogenesis of T2DM and explore possible effective treatment options. Metformin is the well-known oral glucose-lowering agent [8], frequently used as an antidiabetic monotherapy, which decreases plasma glucose levels by several mechanisms, positively influences serum lipid profiles, the process of hemostasis, and exerts anti-inflammatory, antiapoptotic, and antioxidative properties [9, 10]. However, very little is known about the effects of chronic metformin treatment on hypothalamic functions. A recent *in vivo* study is reporting that 4-week metformin administration to either normal or high-fat-fed rats can potentiate the inhibition of hypothalamic AMP-activated protein kinase (AMPK) by leptin but is devoid of any effect of its own [11]. Additionally, another *in vitro* study demonstrated that metformin inhibited AMPK activity in the hypothalamus and neuropeptide Y neurons which explains the appetite suppressing nature of AMPK [12]. Nevertheless, it still remains poorly understood in how many possible ways metformin may influence the hypothalamic functions under the condition of stable hyperglycemia.

Over the last years, hypothalamic inflammation has been linked to the development and progression of obesity; scientists have put their efforts in searching for possible drugs to positively regulate hypothalamic function during T2DM. Short-chain fatty acids (SCFAs), such as propionic acid (PA) and other gut metabolites, were shown to have “anti-obesity properties” by ameliorating fasting glycaemia, body weight, and insulin tolerance in animal models [13]. Moreover, PA and other SCFAs demonstrated anti-inflammatory properties by inhibiting LPS-induced tumor necrosis factor-alpha (TNF-*α*) release from human blood-derived neutrophils and may have beneficial effects on adipose tissue inflammation [14, 15]. Interestingly, although PA produces and accumulates mostly in the gut, it can easily cross the gut-blood and blood-brain barriers (BBB) and gain access to the central nervous system affecting among others and hypothalamic regions by inducing intracellular acidification [16] and alteration of neurotransmitter releases, thus influences neuronal communication and behavior [17]. Furthermore, PA has been demonstrated to modulate the expression of genes, related to the pathogenesis of autism spectrum disorders (ASDs), including those involved in regulation of neurotransmitters, neuroplasticity, neurodevelopment, neuronal cell adhesion molecules, inflammation, oxidative stress, lipid metabolism, and mitochondrial function [16, 18, 19]. However, some controversial effects of PA have been reported in the neurotoxic context [20] and after its short-term intracerebroventricular infusion reversible hyperactive and repetitive behavior was observed [19]. Moreover, it was shown that intraventricular infusions of PA led to a reversible repetitive dystonic behavior, hyperactivity, turning behavior, retropulsion, caudate spiking, and the progressive development of limbic kindled seizures. An increase in oxidative stress markers and glutathione S-transferase activity associated with a decrease in glutathione and glutathione peroxidase activity was found in brain homogenates of PA-treated rats [21]. Additionally, propionic acid, a major SCFA, produced by ASD-associated gastrointestinal bacteria and also a common food preservative, exerts reversible behavioral, electrographic, neuroinflammatory, metabolic, and epigenetic changes closely resembling those found in ASD when administered to rodents [19, 22].

Moreover, it has been shown that fatty acids play an important role in the regulation of ER stress-induced apoptosis in different cell types. Researchers revealed that acetic and propionic acids reduced ER stress on bovine mammary epithelial cell culture [23]. However, direct effects of PA on ventromedial hypothalamic (VMH) neurons under T2DM-induced ER stress remain incompletely studied. Therefore, it might be perspective to elucidate the role of PA in the context of the gut-brain axis as well as in relation to changes in the UPR system of hypothalamus of T2DM animals.

Thus, the present study is aimed at exploring the state of the UPR signaling and astrocyte/microglia crosstalk in the VMH and investigating the impact of metformin and PA in order to interpret the possible neuroprotective effects of PA. In this study, T2DM rats were utilized to determine the levels of the 78 kDa glucose-regulated protein (GRP78), protein *kinase* R-*like endoplasmic reticulum kinase* (PERK), activating transcription factor 6 (ATF6), inositol requiring enzyme 1*α*/*β* (IRE1), ionized calcium-binding adaptor molecule 1 (Iba1), *glial fibrillary acidic protein* (GFAP), and tight junction protein *1 or* zonula occludens-1 (ZO-1) (by RT-PCR and western blot analysis) and assess their distribution in VMH immunohistochemically after metformin and PA treatment and their combined action. Moreover, ultrathin sections of VMH after metformin and PA administration were examined with a transmission electron microscope to visualize the possible morphological changes in the ER.

## 2. Materials and Methods

### 2.1. Animals

The present study used male Wistar rats (176.8 ± 8.3 g) between two and six months of age. Animals were housed in a 12 h light/dark cycle (24 ± 2°C, 65 ± 5% humidity) and were fed a standard, balanced rodent diet and water *ad libitum*. All experimental procedures with animals were carried out in an accordance with national guidelines and international laws concerning animal welfare: “European Convention for the protection of vertebrate animals used for experimental and other scientific purposes” (Strasbourg, 1986), “Bioethical expertise of preclinical and other scientific research conducted on animals” No. 3447-IV (Kyiv, 2006). The protocol of experiments on rats was approved by the Bioethics Committee of the Bogomolets National Medical University (Protocol No. 123 from 23/12/2019).

### 2.2. Experimental Design and Groups

Animals were acclimated for 1 week. Each experimental group included 12 animals: (1) the control group; (2) the group with experimentally induced T2DM by high-fat diet (HFD) for 3 months followed by a single injection of streptozotocin (STZ, 25 mg/kg of b.w.); (3) the group that received antihyperglycemic agent metformin dissolved in water for injection (GLUKOFAGE, Merck Sante, France) at a dose 60 mg/kg of b.w., for 14 days, orally on the background of T2DM; (4) the group that received sodium salt of propionic acid (PROPICUM®, Flexopharm Brain GmbH & Co, Germany) dissolved in water for injection at a dose 60 mg/kg of b.w., for 14 days, orally on the background of T2DM; and (5) the group that received concurrently metformin (60 mg/kg of b.w., for 14 days, orally) and sodium salt of propionic acid (60 mg/kg of b.w., for 14 days, orally) on the background of T2DM. The sodium salt of propionic acid was provided by Prof. Nina Babel (Center for Translational Medicine and Immune Diagnostics Laboratory, Medical Department I, Marien Hospital Herne, University Hospital of the Ruhr University Bochum, Herne, Germany).

**Figure 1:**
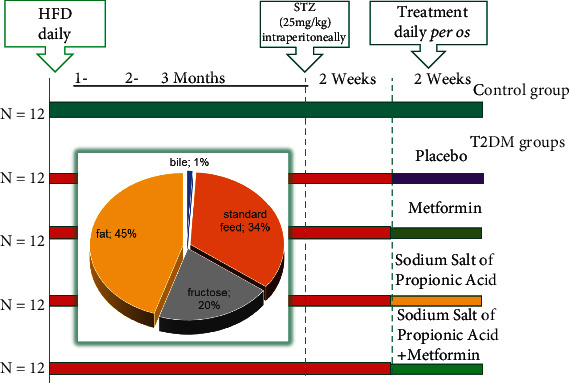
Schematic representation of the experimental design and timescale. Each experimental group included 12 animals: (1) the control group; (2) the group with experimentally induced T2DM; (3) the group that received antihyperglycemic agent metformin (GLUKOFAGE, Merck Sante, France) at a dose 60 mg/kg of b.w., for 14 days, orally on the background of T2DM; (4) the group that received sodium salt of propionic acid (PROPICUM®, Flexopharm Brain GmbH & Co, Germany) at a dose 60 mg/kg of b.w., for 14 days, orally on the background of T2DM; and (5) the group that received concurrent metformin (60 mg/kg of b.w., for 14 days, orally) and sodium salt of propionic acid (60 mg/kg of b.w., for 14 days, orally) on the background of T2DM. T2DM was developed after 3 months of high-fat diet (HFD) with the following single injection of STZ (25 mg/kg of b.w.) according to the protocol [25].

The general scheme of animal experimental design is shown in Figure 1. Experimental T2DM in rats was developed according to the standard protocol that was described previously [24, 25]. Particularly, animals were fed a homogenous HFD mixture: thoroughly grounded standard rodent feed (34%) and premelted fat from lard (45%), medical bile acids (1%, for natural emulsification of fat in the intestine of rats and improvement of its absorption by enterocytes), and dry fructose (20%). Administration of STZ was performed once intraperitoneally after 3 months of HFD, and after injection, the animals were transferred to a standard rodent feed and observed for 2 weeks. After this period, all experimental animals with confirmed T2DM were randomly divided into 4 groups to treat them with placebo, metformin, PA, and metformin+PA, as it was described above. The administration of agents was done *per os* using a stainless-steel feeding cannula. In the placebo group with T2DM, we treated rats with water at a similar volume.

After two-week treatment, rats were sacrificed by decapitation under deep anesthesia with ether. Blood samples were collected for serum separation for glycosylated hemoglobin (HbA1c) determination and then centrifuged (+4°C, 3000 rpm) for 15 min, and serum samples were stored at -80°C until measuring.

### 2.3. Intraperitoneal Insulin Tolerance Test

Intraperitoneal insulin tolerance test (ipITT) was performed at the end of the HFD treatment period. Rats received glucose solution (2 g/kg of body weight) orally after overnight fasting for 14 h. Then, rats were given an intraperitoneal injection with an insulin (Actrapid, Novo Nordisk Pharmaceutical Co., Denmark) at a dose of 0.75 U/kg of body weight. Blood glucose level was monitored at 0, 15, 30, 45, 60, and 90 min after insulin injection by a glucose meter (Roche, Mannheim, Germany).

### 2.4. Measurement of Glycosylated Hemoglobin

The content of HbA1c was determined by the method of ion-exchange chromatography-spectrophotometry [26] using a standard kit (Bio Systems, Spain). The blood samples were collected in tubes with EDTA. 50 *μ*l of the whole blood and 5 ml of distilled water were mixed, erythrocytes were lysed, and then, the mixture was applied to a microcolumn from a kit. The elution was performed with the filling buffer according to the manufacturer's protocol. The *eluates* containing the HbA1c fraction were collected, and their optical density was measured spectrophotometrically at the wavelength of 415 nm. The optical density of the hemolysate sample with total hemoglobin was also determined, and the results were expressed as a percentage (%) of the HbA1c fraction to the total hemoglobin.

### 2.5. Slice Preparation and Electron Microscopy

Rat VMH samples were obtained and fixed in 2.5% glutaraldehyde for 4 hours and Millonig's phosphate buffer (рН 7.4). Additionally, the tissue was fixed with 1% osmium tetroxide for 1 hour. After that, brief washing of slices for 20 min in distilled water was performed; therefore, dehydration in a graded series of ethanol (70%, 80%, 90%, and 100%) and acetone was done. Then, slices were poured into a mixture of epon-araldite (Epoxy embedding medium, #45345-250ML-F, Epoxy embedding medium, hardener DDSA, #45346-250ML-F, and Araldite, #10951-250ML, Sigma, USA). Semithin and ultrathin sections were cut with an ultramicrotome “LKB III” (Sweden). To confirm that we worked with the required brain area, semithin sections (2-3 *μ*m) were stained with methylene blue according to the Hayat method [27]. Ultrathin sections (600-900 Å) were contrasted with 2% uranyl acetate solution and lead citrate. Sections were examined using a transmission electron microscope “PEM-125” (Ukraine) with a magnification of 6000-20000x.

The ImageJ software was used to calculate the areas of the ER membranes and the ER cisterns in each cell. The relative ratio between the areas of ER cisterns to the ER membranes was also determined. The sum of the relative areas of ER membranes and cisterns was considered an overall fraction of ER in the cell. In addition, we calculated the ratio of the area of the perinuclear space to the length of the perimeter of the cell nucleus.

### 2.6. RNA Isolation, cDNA Synthesis, and Real-Time PCR Analysis

The ventromedial hypothalamus samples with a mean weight of 0.03 g were collected, and total RNAs were extracted by GeneJET RNA Purification Kit (Thermo Fisher Scientific Inc., USA). mRNA concentration and purity were measured by DeNovix DS-11 FX+ (DeNovix Inc., USA) at 230, 260, and 280 nm wavelength to determine the OD260/280 and OD260/230 ratio. RNA templates were treated with RNAse-free DNAse I (Thermo Fisher Scientific Inc., USA) to remove trace amounts of DNA. Purified RNA from tissue samples was converted to cDNA by a reverse transcription process using the RevertAid First Strand cDNA Synthesis Kit (Thermo Fisher Scientific Inc., USA). The cDNA samples were used as templates for real-time PCR analysis, which was performed on 7500 Real-time PCR System **(**Life Technologies Corporation, USA). Target genes were amplified for 40 cycles using Maxima SYBR Green/ROX qPCR Master Mix (Thermo Fisher Scientific Inc., USA). A two-stage RT-PCR amplification reaction was performed under the following conditions: 95°C for 5 min, followed by 40 cycles at 95°C for 15 s, and at 60°C for 50 s. The primer sequences were designed using the Primer-BLAST software; they are presented in Table 1. *β-Actin* was used as a reference gene. Data were calculated as the fold change relative to control using the *ΔΔ*Ct method. Duplication was used to reduce the variability within samples.

### 2.7. Protein Extract Preparation and Western Blot Analysis

Target protein levels were assessed by western blot analysis. Total protein extracts were prepared from frozen hypothalamus samples using standard protocol with homogenizing buffer RIPA (20 mM Tris-HCl, pH 7.5; 150 mM NaCl; 1% Triton X-100; 1 mM EGTA; 0.1% SDS; 1% sodium deoxycholate; and 10 mM sodium pyrophosphate). VMH samples (0.03 g) were lysed for 20 min in RIPA (1 : 9) in the presence of protease inhibitor cocktails (Sigma, USA) and then centrifuged for 20 min (14000 × g) at +4°C, and the pellets were discarded. Protein concentration of supernatants was determined by the Stoscheck method [28], and equal amounts of protein (50 *μ*g per track) from each sample were loaded onto 10-15% resolving gel for electrophoresis (depending on the molecular weight of proteins of interest). Then, proteins were transferred onto a nitrocellulose membrane (#HATF00010, Immobilon-NC Transfer Membrane, 0.45 *μ*m pore size, Merck Millipore, USA). Membranes were blocked with 5% nonfat milk in phosphate-buffered saline (PBS) plus 0.05% Tween-20 (PBST) for 1 h followed by an incubation overnight at +4°C with primary antibodies against GRP78 (1 : 1000, #PA5-34941, Thermo Fisher Scientific, USA), PERK (1 : 800, #PA5-79193, Thermo Fisher Scientific, USA), ATF6 (1 : 1000, #PA5-85935, Thermo Fisher Scientific, USA), IRE1 (1:500, #PA5-20190, Thermo Fisher Scientific, USA), ZO-1 (1 : 1000, #61-7300, Thermo Fisher Scientific, USA), GFAP (1 : 1000, sc-9065, Santa Cruz Biotechnology, USA), and tubulin (1 : 1000, T5168, Sigma-Aldrich, USA) in PBS supplemented with 0.1% (vol./vol.) Tween-20 and 5% (wt./vol.) nonfat milk. Primary-antibody-bound membranes were then incubated with HRP-conjugated secondary antibodies: anti-rabbit IgG (1 : 4000, #A0545, Sigma-Aldrich, USA) and anti-mouse IgG (1 : 5000, #ab97057, Abcam, UK). Thereafter, enhanced chemiluminescence was performed for band visualization with chemiluminescent agents: p-coumaric acid (Sigma-Aldrich, USA) and luminol (Sigma-Aldrich, USA). The relative levels of GRP78, PERK, ATF6, ZO-1, Iba-1, and GFAP were normalized to that of tubulin. *The immunoreactive bands were quantified with Gel-Pro Analyzer32, v3.1.*

### 2.8. Immunohistochemistry and Quantification

Immunohistochemical study was performed using polyclonal antibodies against GRP78 (1 : 500, #PA5-34941, Invitrogen, USA) and the Iba1 (1 : 200, #MA5-27726, Invitrogen, USA). Visualization was made using 3,3′-diaminobenzidine (DAB) and the EnVision FLEX detection system (Dako, Denmark).

**Figure 2:**
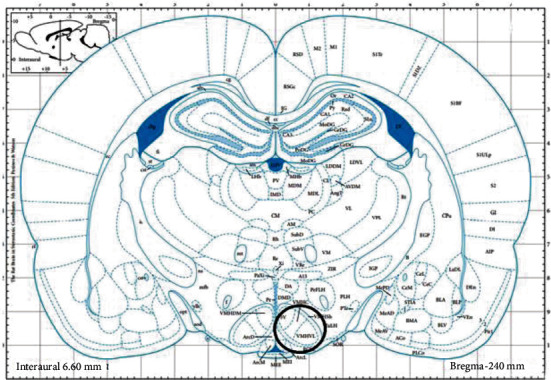
Schematic representation of the brain region (black circle) obtained for immunohistochemical studies. Sections 2.04 and 3.0 mm posterior to Bregma. VMHC: ventromedial hypothalamic nucleus, central part; VMHVL: ventromedial hypothalamic nucleus, ventrolateral part (from Paxinos G, Watson C: The Rat Brain in Stereotactic Coordinates, ed 2. Sydney: Academic Press, 1986).

After euthanization of rats, transcardial perfusion was performed with 0.1 M PBS (pH 7.4) followed by the 4% paraformaldehyde in PBS. The brain was dissected and postfixed in 4% paraformaldehyde overnight, and after that, tissue samples were dehydrated in elevated concentrations of ethanol and embedded in paraplast (Leica-Paraplast Regular, 39601006, Leica Biosystems Inc., USA) according to the standard procedure. Ventromedial hypothalamus sections of 5 *μ*m thick were obtained using a microtome Microm HM360 (Microm International GmbH, Germany) and mounted HistoBond®+ adhesive microscope slides (Marienfeld GmbH & Co. KG, Germany). All brain sections were within stereotaxic coordinates—2.04…3.0 mm relative to Bregma (Figure 2).

Slices were deparaffined in xylene and then rehydrated in an ethanol solution of decreased concentration (100%, 95%, 80%, and 70%; 2 min each step) and 2 steps of distilled water (2 min each). Antigen unmasking was performed in 10 mM sodium citrate buffer (pH 6.0) at the temperature of 98°C for 30 minutes. After cooling to 65°C, the slides were washed for 1 min in 3 volumes of EnVision™ FLEX washing buffer (1 : 20 dilution). The slides were removed from the washing buffer, and endogenous peroxidase activity was blocked with the *EnVision FLEX peroxidase*-blocking reagent solution for 2 min. Sections were incubated for 20 min in the GRP78 and the Iba1 antibodies at room temperature, and then, after washing, sections were incubated with secondary HRP-conjugated antibody from the kit (EnVision FLEX/HRP) for 20 min, and the complex was visualized using a mix of DAB (EnVision DAB+ Chromogen) with EnVision FLEX substrate buffer for 10 min at room temperature. Nuclei were stained with Gill III hematoxylin. Mouse serum was substituted for the antibody as a negative control.

Brain sections were examined using an Olympus microscope BX51 and a digital camera Olympus C4040ZOOM with the software Olympus DP-Soft 3.2. Semiquantitative evaluation of the immunohistochemical reaction was performed, and the intensity of staining was calculated as follows: grade 0 for no reaction or focal weak reaction, grade 1 for intense focal or diffuse weak reaction, grade 2 for moderate diffuse reaction, grade 3 for intense diffuse reaction, and grade 4 for intense labeling. The obtained digital data were processed by standard statistical methods. The Student *t*-test was used to assess the significance of differences in mean values when comparing groups. *p* < 0.05 was considered statistically significant.

### 2.9. Statistical Analysis

The data distribution was analyzed using the Shapiro-Wilk test. For normally distributed data, statistical differences between the groups were analyzed by the one-way ANOVA test with the following Tukey post hoc test. The difference was considered to be statistically significant when *p* < 0.05. The Pearson's correlation coefficient (*R*) was calculated with a *p* value of 0.05 (95% confidence interval). All data were obtained based on two or three independent experiments and expressed as mean ± SEM. Statistical analysis was performed using “IBM SPSS Statistics for Windows, version 23” (IBM Corp., Armonk, N.Y., USA).

## 3. Results

### 3.1. Effects of Metformin and PA on Animal Body Parameters, Peripheral Glucose Homeostasis, and Insulin Sensitivity

To confirm the development of MS after HFD, we performed an intraperitoneal glucose tolerance test and measured animal body parameters (weight, body length, and waist) and the levels of glucose and HbA1c in the serum of animals.

We found that after 3.5 months from the start of experiment, body weight of T2DM rats was significantly higher by 1.65-fold (*p* = 0.0003), and the length and waist were also increased (by 15%, *p* = 0.013 and 35%, *p* = 0.00008, respectively) compared with control animals (Table 2). Administration of metformin and PA, as well as their combination, had no significant effects on body weight compared with the T2DM group; however, the body weight in metformin group (by 67%, *p* = 0.0002), in PA group (by 63%, *p* = 0.0002), and in metformin+PA group (by 51%, *p* = 0.004) was higher compared with control animals. Administration of PA and a combination of metformin and PA did not influence the length and waist parameters compared with T2DM animals. Thus, animals in the T2DM group demonstrated a significant increase in weight and body parameters compared with the control group. This reflects the development of obesity, which is one of the side consequences of hyperglycemia and dyslipidemia in T2DM.

To assess the peripheral glucose homeostasis and further confirm the development of stable hyperglycemia, the levels of glucose and glycosylated hemoglobin in the serum were measured and the ipITT was performed. We found that 4 weeks after the injection of STZ, fasting blood glucose exceeded the control level by 1.97-fold (*p* = 0.004, Table 2) reflecting the state of hyperglycemia. After treatment with metformin for 2 weeks, the glucose level did not change, and it was still higher by 1.96-fold (*p* = 0.004) in comparison with the control. The blood glucose levels in the PA group were higher than in control animals by 2.17-fold (*p* = 0.00028). The animals from the metformin and PA group demonstrated the glucose level that was elevated by 1.87-fold (*p* = 0.01) compared with control animals and lowered by 14% (*p* = 0.038) compared with PA administration.

The next important marker of hyperglycemia we assessed was the level of HbA1c. We observed a similar to glucose level fluctuations when measuring HbA1c content and found that it was higher in T2DM rats by 1.74-fold (*p* = 0.049) and by 1.70-fold (*p* = 0.067) in the metformin group vs. control. However, PA administration had a slight increasing effect on HbA1c content not only compared with control animals (2.10-fold, *p* = 0.001), but also when compared with the T2DM (by 21%, *p* = 0.038) and the metformin group (by 23.9%, *p* = 0.039). Surprisingly, treatment with metformin and PA had the lowering effect on HbA1c serum level by 13% vs. the T2DM group (*p* = 0.046) and by 36.6% vs. the PA group (*p* = 0.029); however, it remained higher than in control animals (1.54 times, *p* = 0.032).

Thus, persistent hyperglycemia and elevated HbA1c level as stable and accurate biomarkers have verified the development of experimentally induced T2DM in rats.

**Figure 3:**
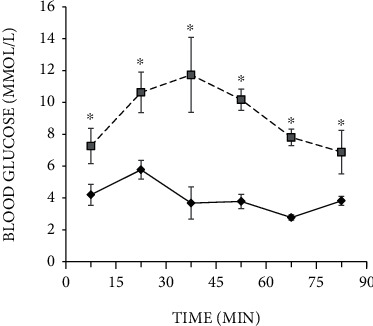
Intraperitoneal insulin tolerance test on control rats (*n* = 12, solid line) and rats with T2DM (*n* = 12, dashed line) before administration of substances. Blood samples were collected from the tail at the indicated time points and analyzed for glucose concentration (mmol/l). Values are means ± SEM. ^∗^*p* < 0.05 compared with control.

Next, to determine the sensitivity of insulin-responsive tissues, we performed intraperitoneal insulin tolerance test based on the measurement of glucose remaining in the circulation over time after an intraperitoneal insulin injection (Figure 3). As we expected, at the time point of 15 min after the injection of insulin, blood glucose level of control animals increased by 1.3-fold (*p* = 0.042) compared with the initial value (4.25 mmol/l) and it was the maximum point (5.77 ± 0.29 mmol/l). Then, the glucose level decreased to a value of 1.56-fold (*p* = 0.001 compared with 15 min point) and significantly altered on the 45^th^ minute. At 60 minutes after the insulin injection, the glucose level was minimal (2.7 ± 0.08 mmol/l), and then, after 15 minutes, the level increased to 3.8 ± 0.14 mmol/l.

In the T2DM group, the initial glucose level was 7.26 ± 0.45 mmol/l, and within 15 minutes after the insulin injection, it was increased by 1.46-fold (*p* = 0.003) to 10.63 mmol/l; after 30 min, it was reached a maximum value—11.73 ± 0.96 mmol/l. Then, the glucose level progressively decreased to 10.17 ± 0.27 mmol/l on the 45^th^ minute and to 7.8 ± 0.21 mmol/l on the 60^th^ minute of an observation. 90 min after injection, the blood glucose level in T2DM rats was minimal (6.88 ± 0.55 mmol/l). The obtained data demonstrated significant inhibition of insulin secretion in response to glucose load in the T2DM group compared with the control group.

Thus, rats from the T2DM group exhibited impaired glucose tolerance and insulin sensitivity, which confirmed the impairments in glucose homeostasis.

### 3.2. Ultrastructural Changes of ER in the VMH of Rats with T2DM after Metformin and PA Administration

The endoplasmic reticulum is a dynamic intracellular organelle involving in maintenance of cellular homeostasis and stress response under physiological and pathological conditions. Increasing evidence has shown a strong interconnection between ER stress and the pathology of obesity and T2DM that may lead to leptin and insulin resistance. To investigate the possible changes in the VMH architectonics and ER ultrastructure in the hypothalamus of rats with T2DM and the influence of the metformin and propionic acid administration, the examination of VMH cytoarchitectonics by electronic microscopy and an assessment of morphometric parameters of ER were performed.

**Figure 4:**
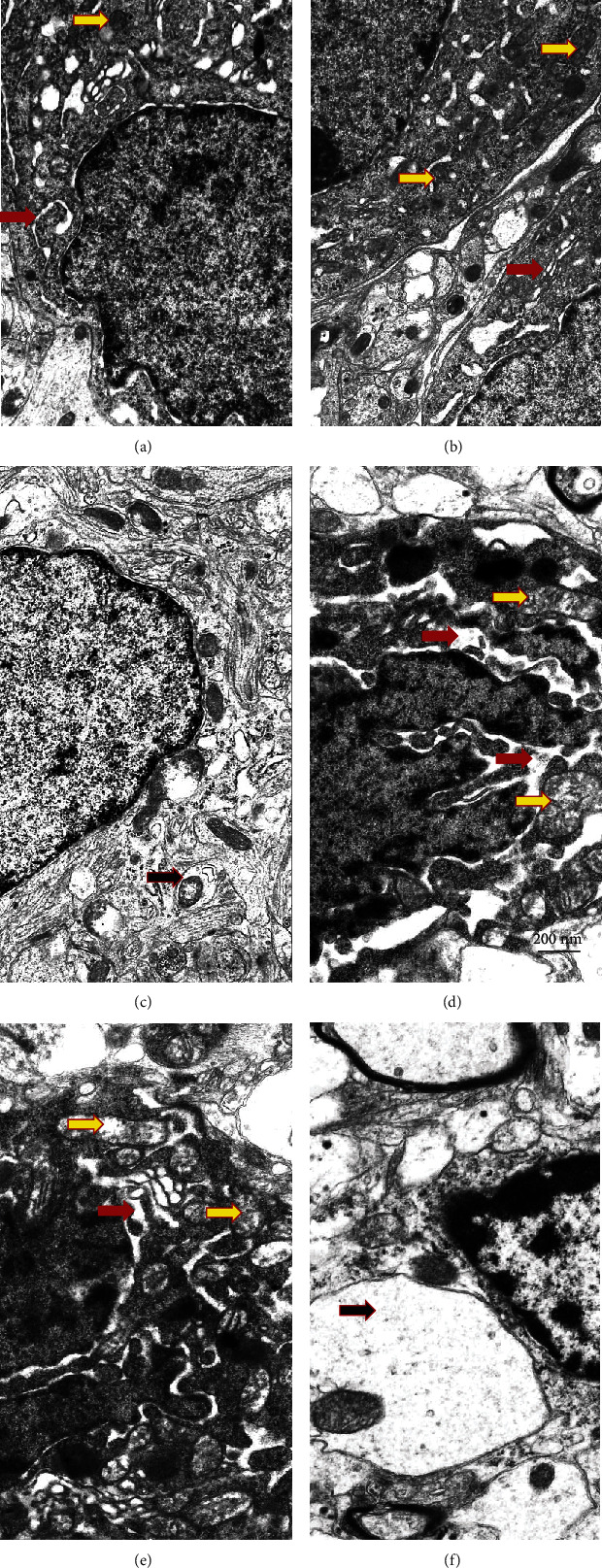
Ultrastructural changes of VMH neurons and glial cells were assessed by electron microscopy observations. Gallery of micrographs obtained by SEM (*n* = 12 neurons for each group): (a–c) control rats; (d–f) rats from the T2DM group. Representative images of neurons are presented on (a, b, d, e) and glial cells on (c, f). The red arrows indicate the rough endoplasmic reticulum, yellow arrows—mitochondria, and black arrows—neuropil swelling. Scale bar: 200 nm.

Electron microscopic examination revealed that most of the neurons in VMH of T2DM animals had increased electron density of the cytoplasm and nucleus compared with control (Figure 4). Compared with control (Figures 4(a) and 4(b), red arrows), in the T2DM group, tubules of *rough* endoplasmic reticulum in most neurons were partially fragmented and enlarged (Figures 4(d) and 4(e)). A small number of attached to the ER tubules ribosomes were found; however, the most of them were “detached” from the tubules and located in the cytoplasm, where they formed cluster-like polysomes. Such cluster-like structures may be responsible for an increased electron density of neurons. In most observed rat neurons in the T2DM group, we found an accumulation of fragmented mitochondria with signs of cristae *destruction* (yellow arrow) and matrix swelling. Mitochondria with destroyed cristae underwent the process of autophagy and formed mitophagosomes. Most nuclei were pyknotically altered with signs of chromatin condensation and demonstrated the appearance of deep invaginations of the nuclear membrane; additionally, nuclei fragmentation in a part of neurons was observed. Neurons with signs of apoptosis and dendritic loss predominated in the T2DM group. Swelling of synaptic terminals with loss of vesicles with neurotransmitters was found in the neuropil. The morphopathological changes of glial cells in T2DM rats (Figure 4(f)) were detected compared with control (Figure 4(c)); there was swelling of the cytoplasm (Figure 4(f), black arrow), and a reduced number of organelles was observed.

**Figure 5:**
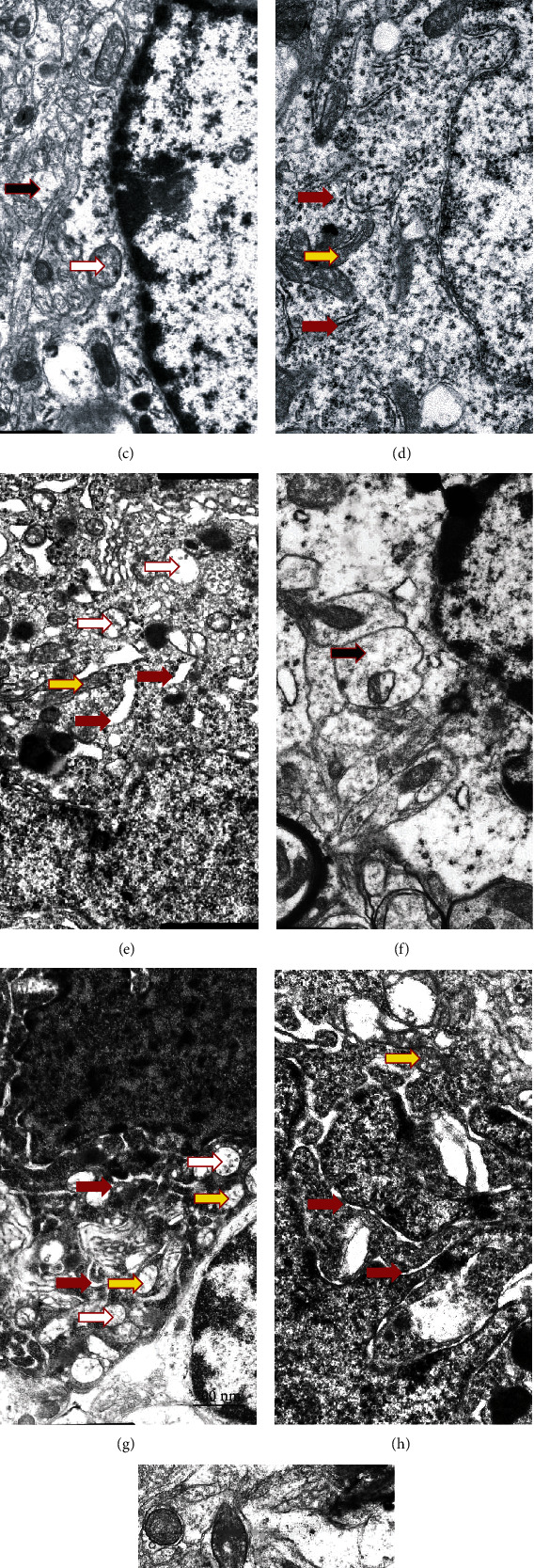
Ultrastructural changes of VMH neurons and glial cells were assessed by electron microscopy observations. Gallery of micrographs obtained by SEM (*n* = 12 neurons for each group): (a–c) metformin group; (d–f) PA group; (g–i) metformin and PA group. Representative images of neurons are shown on (a, b, d, e, h, g) and glial cells on (c, f, i). The red arrows indicate the rough endoplasmic reticulum, yellow arrows—mitochondria, white arrows—autophagosomes, and black arrows—neuropil swelling. Scale bar: 200 nm.

The administration of drugs slightly improved the morphological signs of damage in the nervous tissue (Figure 5). The use of metformin led to a clearly visible accumulation of lipofuscin granules in the cytoplasm of neurons and an increase in the number of lysosomes and autophagosomes (Figures 5(a)–5(c), white arrows). The volume of the ER cisterns was reduced compared to the T2DM group. Ribosomes were located on the surface of the ER membranes. The structure of mitochondria was more preserved than in the T2DM group. Additionally, we observed the presence of reactive astrogliosis, characterized by an abnormal increase in the number of astrocytes, their enhanced proliferation, and hypertrophy. In the VMH of rats from the metformin group, swelling of myelinated *fibers*, an accumulation of autophagosomes in axoplasm and damage of the myelin sheath, was detected.

Administration of PA to rats with T2DM caused the corresponding changes of neuron ultrastructure compared with the metformin group (Figures 5(d)–5(f)). We observed that the volume of ER cisterns was well preserved in neurons, with the characteristics of cisterns that did not almost differ from the control. Tubules of the rough endoplasmic reticulum were well preserved with clearly visible ribosomes located on its surface without accumulating in the cytoplasm, as it was shown in T2DM. Autophagosomes of both types—primary with a double-layer membrane and secondary with a single-layer membrane and with the cellular detritus, were observed, which might be a sign of the accumulation of abnormally folded proteins. PA administration led to a decrease in the number of apoptotic dark neurons with pyknotically altered nuclei compared with T2DM (Figures 4(d) and 4(e)). In a small number of cells, a partial enlargement of the perinuclear space was observed. Chromatin was homogeneously distributed within the nucleus with the predominance of its active form—euchromatin. Astrocytic glia demonstrated no signs of swelling. Thus, according to our observations, PA probably in more extent than metformin prevented diabetes-induced alterations of VMH cytoarchitectonics.

When examining the combination of metformin and PA, we found an increase in the quantity of microglial cells and an almost 2-fold reduction of the percentage of light neurons compared with the control group (Figures 5(g)–5(i)). In addition, when using a concurrent administration of metformin and PA, there was a significant elevation in the number of preapoptotic and apoptotic neurons that in turn may activate microglia. In light cells, a significant number of ER cisterns and tubules with a small number of ribosomes were visualized. Cisterns and vesicles of the Golgi apparatus were widespread and well visualized in dark cells (Figure 5(h)). Mitochondria were swollen partially, and an increased number of lysosomes and lipofuscin granules were detected compared with the control group. However, there were a fewer amount of lipofuscin granules compared to metformin administration (Figures 5(b) and 5(i)). The nuclei of light neurons had significantly more invaginations of the nuclear envelope compared to dark cells. Interestingly, we found that astrocytes demonstrated much less pronounced swelling near dark neurons when compared with the T2DM group. The formation of autophagosomes containing cellular detritus was increased in the PA group and in the metformin and PA group that is indicated in Figure 5 by white arrows.

Thus, based on electron microscopy examinations of the VMH samples, it should be noted that in diabetic rats, most neurons had an increased electron density of the cytoplasm due to the formation of polysomes—clusters of ribosomes that were detached from the ER. The destruction of mitochondrial cristae, nuclear pyknosis, the appearance of deep invaginations of the nuclear membrane, nuclear fragmentation, and an increase in the number of apoptotic neurons were detected. The administration of drugs partially prevented ER damage and mitochondrial swelling, reduced the number of apoptotic neurons to a different extent, and enhanced the glial response.

For a more objective assessment of the influence of metformin and PA administration on the background of T2DM on the parameters of the ER structure of the VMH, we carried out a morphological assessment of the ER parameters in the groups (Table 3).

Quantitative measurement of the parameters of the ER showed an enlargement of the ER cisterns in the T2DM group as compared with the control and an increase in their relative area by 2.25-fold (*p* = 0.024). The relative area of the ER membranes did not change in the T2DM group vs. control; thus, the cistern/membrane ratio also increased (*p* = 0.046 vs. control). The total fraction of ER in the neurons of rats with T2DM increased by 19%, and the ratio of the area of the perinuclear space/length of the nucleus perimeter was higher by 44% (*p* = 0.038 vs. control).

The results in Table 3 show that the administration of drugs caused multidirectional changes in the ER parameters. The relative area of the ER membranes progressively decreased in the metformin, PA, and PA and metformin groups by 22% (*p* = 0.048), 33% (*p* = 0.039), and 62% (*p* = 0.019), respectively, compared with the T2DM group. Additionally, metformin reduced the relative area of the ER cisterns in T2DM rats by 36% (*p* = 0.046 vs. T2DM); however, this parameter was 65% higher than in the control (*p* = 0.046). Metformin did not influence the cisterns/membranes ratio compared with T2DM, but it was 2-fold higher compared to the control (*p* = 0.093). The total fraction of ER in the cell and ratio of the area of the perinuclear space to the length of the nucleus perimeter showed no significant changes in the metformin group compared both with control and T2DM rats.

РA administration reduced the cistern/membrane ratio to control value mostly due to concurrent decrease in the areas of ER cisterns (by 40.5%, *p* = 0.055 vs. control, by 316%, *p* = 0.009 vs. T2DM, and by 232%, *p* = 0.026 vs. metformin) and membranes (by 38%, *p* = 0.01 vs. control, by 49%, *p* = 0.009 vs. T2DM, and by 16%, *p* = 0.045 vs. metformin) in VMH neurons. Total fraction of ER in the cell and ratio of the area of the perinuclear space to the length of the nucleus perimeter was also reduced after PA action compared with the control (39%, *p* = 0.045) and T2DM (66%, *p* = 0.03) groups and compared with all the three groups, respectively (by 38%, *p* = 0.03 vs. control, by 49%, *p* = 0.023 vs. T2DM, and by 16%, *p* = 0.045 vs. metformin).

The combined administration of PA and metformin exerted profound lowering effect on the relative area of ER membranes (by 50%, *p* = 0.03 vs. control and by 62%, *p* = 0.019 vs. T2DM) and cisterns (by 53%, *p* = 0.052 vs. control, by 344%, *p* = 0.008 vs. T2DM, and by 253%, *p* = 0.024 vs. metformin) and on the total fraction of ER in the cell (by 50%, *p* = 0.038 vs. control and by 80%, *p* = 0.019 vs. T2DM) and returning the ratio S_cist_/S_membr_ and the ratio of the area of the perinuclear space/length of the nucleus perimeter to the control values.

Thus, an interesting trend was observed under the influence of compounds: after T2DM-induced elevation, three ER parameters (relative area of ER membranes and cisterns and the total fraction of ER in the cell) were lowered to different extent after the treatment with metformin, PA, and their combined exposure.

### 3.3. Effects of Metformin and Propionic Acid on the UPR System in the Hypothalamus of Rats with T2DM

Diabetes-associated perturbation of ER homeostasis results in the development of ER stress that is tightly linked to the functioning of the UPR program. To evaluate the state of the UPR system, which consists of GRP78, PERK, ATF6, and IRE1, we measured the levels of all the components by western blotting and RT-PCR, and additionally, GRP78 distribution in VMH was assessed immunohistochemically.

**Figure 6:**
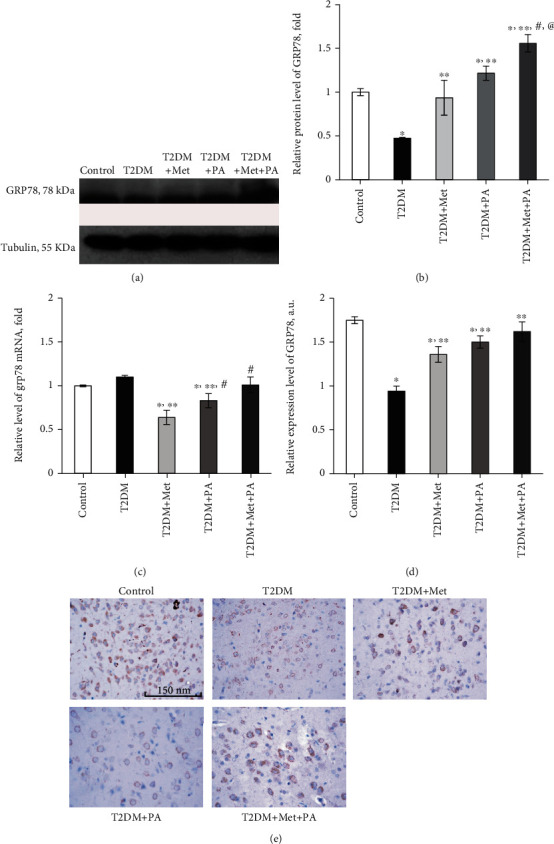
Effects of administration of metformin and PA on the level of unfolded protein response regulator GRP78/BiP. (a) Immunoblotting analysis of GRP78 in rat VMH: representative immunoblots are shown and quantified using tubulin as a loading control for hypothalamus lysates. (b) The bar graphs of GRP78 are presented as means ± SEM (*n* = 6/group). (c) Quantitative polymerase chain reaction (PCR) of *Grp78* in rat VMH: data were normalized to *β*-actin and pooled from three independent experiments (*n* = 6 rats/group). Immunocytochemical analysis of GRP78-positive VMH cells: representative (d) histogram and (e) images are shown. DAB staining was used to visualize GRP78-positive cells; Hematoxylin Gill was used for nuclear staining. Scale bars indicate 150 *μ*m (magnification ×400). All data are shown as means ± SEM; ^∗^*p* < 0.05 vs. control, ^∗∗^*p* < 0.05 vs. T2DM, ^#^*p* < 0.05 vs. metformin administration, and ^@^*p* < 0.05 vs. PA administration.

As it is shown in Figures 6(a) and 6(b), the protein level of GRP78, the UPR master regulator, in the VMH was 2.1-fold lower in T2DM than in the control group (*p* = 0.005), reflecting impaired UPR and possible proteotoxicity, while mRNA *Grp78* level did not change (Figure 6(c)). Metformin administration restored GRP78 protein level to control value and elevated by 1.97 times compared with T2DM (*p* = 0.048) at the background of slightly declined *Grp78* mRNA (by 1.56-fold, *p* = 0.05 vs. control and by 1.72-fold, *p* = 0.04 vs. T2DM). PA administration on the T2DM background increased GRP78 both on the transcriptional and translational levels (protein by 2.56 times vs. T2DM, *p* < 0.001 and by 33%, *p* = 0.045 vs. T2DM group, respectively). Metformin and PA combination restored *Grp78* mRNA level to that observed in the control group; however, GRP78 protein content was elevated by 1.56-fold vs. control (*p* = 0.011), by 3.28-fold vs. T2DM (*p* < 0.001), and by 1.28-fold vs. metformin group (*p* = 0.015).

Immunohistochemical staining of VMH slices with GRP78 antibody confirmed changes observed on the protein level, and images are presented in Figure 6(e). The immunohistochemical study of control VMH slices showed the profound and well-visualized expression of GRP78. In the control group, most neurons showed mild staining of the cytoplasm, including areas with more intense color. Small weakly stained granules were found in neuropil. In animals with T2DM, a sharp decrease in GRP78 expression in VMH neurons was observed with not homogeneous, but granular cytoplasmic staining. These data are in line with the results of western blot analysis that revealed a diabetes-induced decrease in GRP78 level in the ventromedial hypothalamus. In VMH neurons of rats with diabetes mellitus treated with metformin, there was an increase in GRP78 expression compared with diabetic animals. However, GRP78 staining was irregular: in some cases, the content of GRP78 rose up to the control level and was relatively homogeneous; however, in a large part of neurons, GRP78 expression remained at a low level. In the neuropil, a small number of mildly labeled granules and/or small structures of irregular shape were visualized. Treatment with propionic acid caused an increase in GRP78 expression in neurons compared with the metformin group; however, a significant number of cells of this type were poorly stained. Combined treatment with metformin and PA led to a restoration of GRP78 expression in VMH neurons even compared with the control microphotographs. However, in contrast to diffuse cytoplasm staining as it was observed in the control group, the cytoplasm of VMH neurons of rats treated with metformin and PA was clearly granular. In addition, a small number of cells did not express GRP78 protein in this group. Interestingly, some glial cells showed the presence of single chromogenic granules alongside to the nucleus. In addition, after combined treatment with metformin and PA, a small number of dusty immunoreactive granules with a low level of labeling were detected in the neuropil.

**Figure 7:**
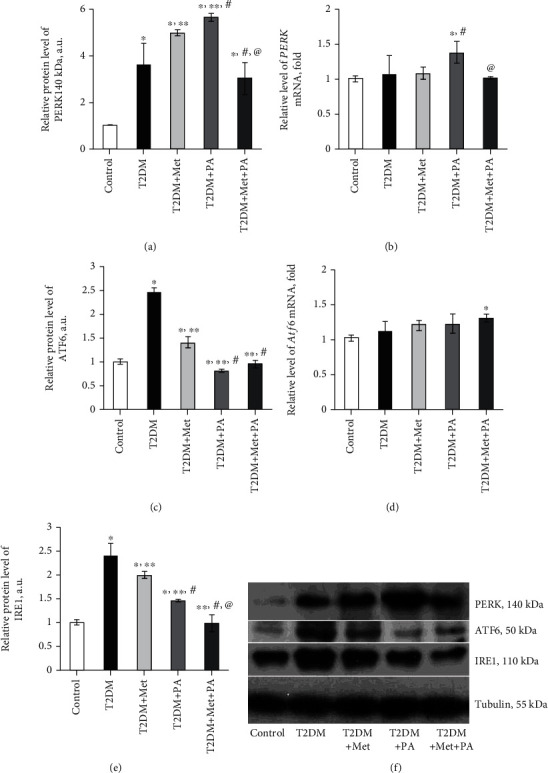
Effects of administration of metformin and PA on the major components of unfolded protein response system—PERK, ATF6, and IRE1. Immunoblotting analysis of PERK, ATF6, and IRE1 in rat VMH: the bar graphs of (a) PERK, (c) ATF6, and (e) IRE1 protein content are presented; (f) representative immunoblots are shown, and data are quantified using tubulin as a loading control for hypothalamus lysates. Quantitative RT-PCR of (b) *Perk* and (d) *Atf6* in rat VMH: data were normalized to *β*-actin and pooled from three independent experiments (*n* = 6 rats/group). All data are shown as means ± SEM; ^∗^*p* < 0.05 vs. control, ^∗∗^*p* < 0.05 vs. T2DM, ^#^*p* < 0.05 vs. metformin administration, and ^@^*p* < 0.05 vs. PA administration.

The next step was to estimate the major components of UPR, a cell-signaling system that readjusts ER folding capacity to restore protein homeostasis. The protein level of the regulator of translation response of UPR and an ER-resident transmembrane protein kinase PERK was elevated by 3.61-fold in the T2DM group (*р* = 0.039 vs. control, Figures 7(a) and 7(f)). Drug administration led to a further increase in PERK content vs. control: metformin—by 4.98-fold (*p* = 0.008) and PA—by 5.64-fold (*p* = 0.001). In contrast, combined metformin+PA administration had no effect on PERK protein level compared with the T2DM group, but it was 3.01-fold higher than in control (*p* = 0.002). Intriguingly, when we studied *Perk* mRNA level (Figure 7(b)), we observed a stable mRNA expression in control, T2DM, metformin, and metformin+PA groups with no statistically significant differences between them. However, PA administration showed a tendency to increase *Perk* mRNA level by 38% compared with control (*p* = 0.045).

The next UPR component, the level of which we determined, was ATF6, an ER-localized transmembrane protein and a major UPR sensor. A 2.44-fold increase in ATF6 level was observed in the T2DM group (*p* = 0.02, vs. control, Figure 7(c)), reflecting its enhanced translocation/accumulation in Golgi apparatus upon proteotoxic ER stress. Metformin administration led to a decrease in ATF6 content by 1.76-fold vs. T2DM (*p* = 0.008). Interestingly, PA exerted a more pronounced lowering effect on ATF6 (3-fold, *p* < 0.001 vs. T2DM and 1.23-fold, *p* = 0.05 vs. control), while combined treatment restored ATF6 up to control value (Figure 7(c)). We have also determined *Atf6* mRNA content and observed that changes at the mRNA level were not as significant as at the protein level (Figure 7(d)). There were no statistically significant differences between control, T2DM, metformin, and PA groups; however, the combined treatment caused a tendency to increase *Atf6* mRNA by 1.28 times compared with control (*p* = 0.048).

The last component that required determination was IRE1, an ER-transmembrane protein and an important UPR sensor. The protein level of IRE1 was increased in the T2DM group (2.4-fold, *p* = 0.001), after metformin administration—by 1.99-fold (*p* = 0.021) and after PA treatment—by 1.45-fold (*p* = 0.045) vs. control (Figures 7(e) and 7(f)). Interestingly, combined treatment with metformin and PA normalized IRE1 content to the control value in the VMH samples.

Thus, the misbalance between the UPR system components was observed under diabetes mellitus, which was more pronounced at the protein level than in mRNA, and treatment with metformin and PA partially normalized the ratio between UPR sensors and regulators.

### 3.4. Protective Effect of Propionic Acid on Impairments of Astrocyte/Microglia Crosstalk in Ventromedial Hypothalamus of Rats with Type 2 Diabetes Mellitus

**Figure 8:**
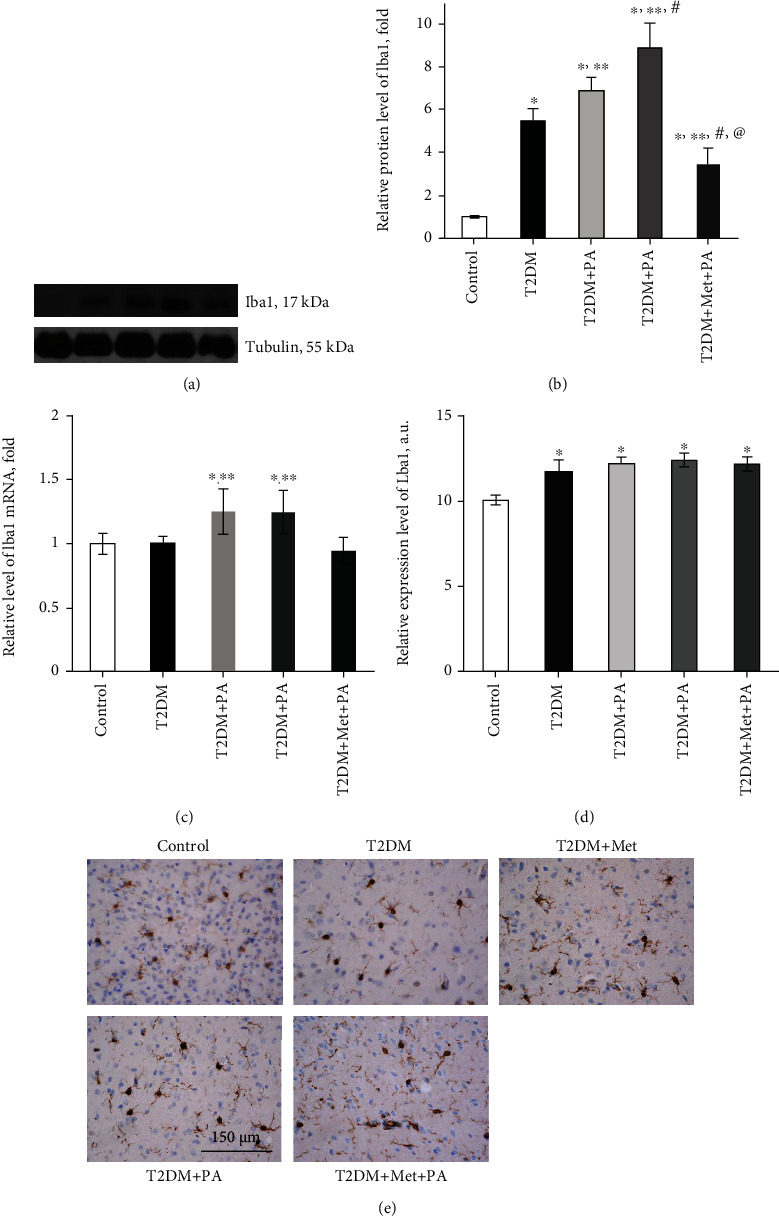
Effects of metformin and PA treatment on the content of microglial marker Iba1. Immunoblotting analysis of the Iba1 in rat VMH: (a) representative immunoblots are shown and quantified using tubulin as a loading control for hypothalamus lysates. The bar graphs of the (b) Iba1 protein level are presented as means ± SEM (*n* = 6/group). Quantitative RT-PCR of the (c) *Iba1* in rat VMH: data were normalized to *β*-actin and pooled from three independent experiments (*n* = 6 rats/group). Immunocytochemical analysis of the Iba1-positive VMH cells: representative (d) histogram and (e) images of representative VMH sections showing the Iba1-positive microglial cells are shown. DAB staining was used to visualize the Iba1-positive microglial cells; Hematoxylin Gill was used for nuclear staining. Scale bars indicate 150 *μ*m (magnification ×400). All data are shown as means ± SEM; ^∗^*p* < 0.05 vs. control, ^∗∗^*p* < 0.05 vs. T2DM, ^#^*p* < 0.05 vs. metformin administration, and ^@^*p* < 0.05 vs. PA administration.

It is known that hypothalamic dysregulation is one of the underlying mechanisms of abnormal glucose metabolism and T2DM. However, mechanisms of astrocyte/microglia crosstalk in the ventromedial nucleus of the hypothalamus on the background of T2DM remain undiscovered. Since PA is involved in the neuroinflammatory response in rats, therefore our next step was to assess the effect of PA administration on markers of microglia and astrocytes in VMH of T2DM rats. The ionized calcium-binding adaptor molecule 1, Iba1, is considered a microglial marker and a macrophage-specific calcium-binding protein. To investigate the overall contribution of microglia to T2DM-induced hypothalamic dysregulation, we determined the Iba1 protein and mRNA levels and assessed the Iba1 distribution in VMH of experimental animals immunohistochemically. As we can see from Figures 8(a) and 8(b), the Iba1 protein level was significantly increased (5.44-fold) in the T2DM group (*p* = 0.01 vs. control). Metformin administration on the background of T2DM increased the Iba1 protein level by 6.88-fold (*p* = 0.003 vs. control). Interestingly, PA treatment led to the further elevation of the Iba1 content (8.9-fold, *p* < 0.001 vs. control). By contrast, simultaneous PA and metformin treatment declined the Iba1 protein content by 1.97 times compared with metformin (*p* = 0.02) and by 2.54 times compared with PA treatment (*p* = 0.004); however, the Iba1 level was still 3.5-fold higher than in control. Interestingly, when we determined the Iba1 mRNA levels by RT-PCR, we did not observe such pronounced effects as it was on the protein level (Figure 8(c)). A separate administration of metformin and PA exerted a slight increasing effect on the Iba1 mRNA levels (by 1.25-fold in both groups, *p* = 0.04 vs. control), while in the T2DM and metformin+PA groups, the *Iba1* mRNA level was as in the control group.

In addition, a semiquantitative immunohistochemical assessment of the Iba1 distribution in VMH showed a slight tendency to increase in the T2DM group and after compound administration (Figures 8(d) and 8(e)). Animals of the control group showed a moderate number of the Iba1-positive cells in the VMH (Figure 8(e)). Microglial cells contain compact cell nuclei surrounded by a narrow line of cytoplasm showed immunoreactivity to the Iba1 protein. The level of the Iba1 expression in both cell bodies and *processes* was classified as moderate. Under T2DM conditions, a marked increase in the expression of the Iba1 in microglial cells of VMH was observed (Figure 8(e)). At the same time, the average size of microglial cell bodies was noticeably increased, and the level of expression of the Iba1 also increased, however to a greater extent in cell bodies and to a lesser extent—in their *processes*. Visually, there was a slight increase in the number of *processes* of the Iba1-positive cells, which may indicate the ramification process. Administration of metformin led to an increase in the number of microglial processes with the expression of the Iba1 compared with the T2DM group. However, the overall Iba1 expression level in both cell bodies and processes did not differ from the T2DM group (Figure 8(d)). In VMH of rats that received PA, we observed an increase in the level of the Iba1 expression compared with control along with more *hypertrophic microglial cells when* compared with control and T2DM (Figure 8(e)). Cell processes were marked more contrastingly than in the T2DM group. In animals that simultaneously received metformin and PA, a slight decrease in the expression of the Iba1 in VMH was observed, mostly in the bodies of microglial cells (Figure 8(e)). Visually, there was no difference in the number of immunostained processes between 3 groups: with metformin treatment, with PA administration, and with combined metformin and PA exposure.

**Figure 9:**
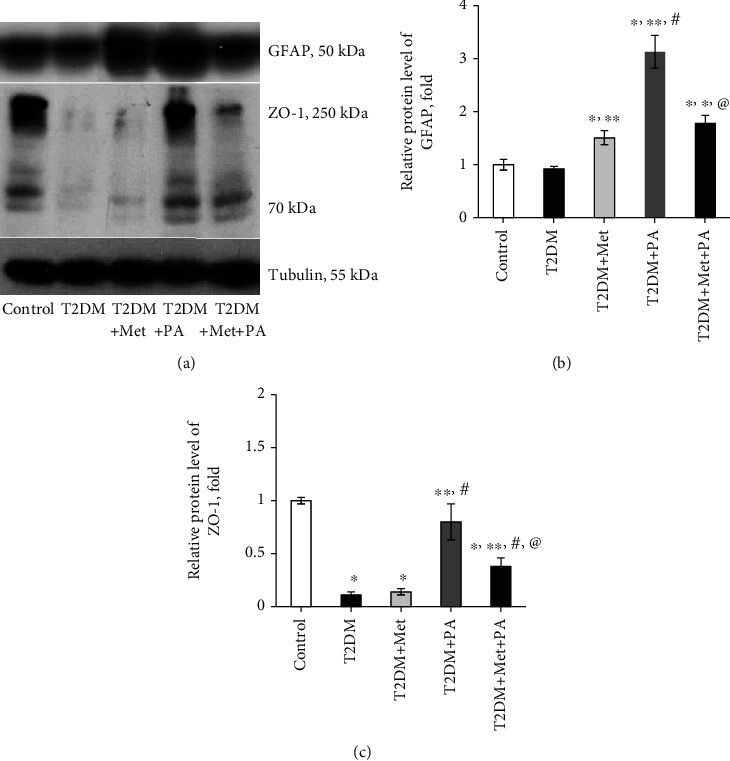
Metformin and PA influence on the level of astrocytic marker GFAP and tight junction protein ZO-1. Immunoblotting analysis of GFAP and ZO-1 in rat VMH: (a) representative immunoblots are shown and quantified using tubulin as a loading control for hypothalamus lysates. The bar graphs of (b) GFAP and (c) ZO-1 protein levels are presented as means ± SEM (n =6/group). Data are shown as means ± SEM; ^∗^*p* < 0.05 vs. control, ^∗∗^*p* < 0.05 vs. T2DM, ^#^*p* < 0.05 vs. metformin administration, and ^@^*p* < 0.05 vs. PA administration.

The next question we addressed is related to the assessment of the level of GFAP, a main intermediate filament protein in mature astrocytes, which represent another subtype of glial cells in the central nervous system. Interestingly, it was shown that the protein level of GFAP did not change in the T2DM group (Figures 9(a) and 9(b)). Separate drug administration led to an increase in GFAP level in VMH: metformin—by 1.51-fold vs. control (*p* = 0.03) and PA—by 3.13-fold (*p* < 0.001 vs. control, T2DM, and metformin). Combined PA and metformin treatment induced a 1.75-fold decrease in GFAP vs. PA (*p* = 0.0004); however, it was still 1.78-fold higher than in control animals (*p* = 0.048). Thus, an observed drug-induced astrocyte activation may reflect a protective response to T2DM-associated neurological injury and contribute to the restoration of VMH homeostasis.

The last step was to estimate the content of ZO-1—the constitutive tight junction protein playing an important role in maintaining cell integrity (Figures 9(a) and 9(c)). It was shown that ZO-1 is present in the primary culture of astrocytes to form tight junctions [29]. We observed that T2DM-induced changes were associated with dramatically decreased ZO-1 level (by 89%, *p* = 0.002 vs. control, Figure 9(c)). Metformin did not affect the ZO-1 level when compared with the T2DM group, while PA treatment increased it almost to control values. Interestingly, combined treatment of diabetic rats with metformin and PA had an increasing effect on ZO-1 protein level (by 3.5-fold, *p* = 0.05 vs. T2DM); however, ZO-1content was still by 62% lower than in control (*p* = 0.03).

Thus, we demonstrated the presence of T2DM-induced activation of microglia and impairments in cell integrity of VMH. Drug administration caused an astrocyte activation to a different extent; however, a combination of PA and metformin effectively lowered the Iba1 levels and partially restored the ZO-1 content, suggesting their perspective immunomodulatory and neuroprotective effects.

## 4. Discussion

Obesity, MS, and T2DM are becoming the 21^st^ century's most serious health problem. Animal studies may help to identify the mechanisms that underlie the adverse impact of diabetes on hypothalamic functions and may also help to screen potential treatment options. In our study, animals in the T2DM group demonstrated a significant increase in weight and body parameters compared with the control rats, which reflect the development of obesity that was in agreement with other studies [30, 31]. An elevation of blood glucose in the serum of T2DM rats was evidence of persistent hyperglycemia, and an elevated HbA1c level was an accurate biomarker that verified the development of experimentally induced diabetes in rats. Finally, impaired glucose tolerance and insulin sensitivity (based on ipITT) confirmed impairments in peripheral glucose homeostasis. After T2DM modeling, we have chosen one of the SCFAs—PA as a potential treatment drug, and metformin as a “gold standard” and drug of choice to treat patients with T2DM. Related to PA choice, first of all, it has demonstrated anti-inflammatory and antiobesity properties of PA in animal models. Secondly, although mostly accumulating in the gut, PA can easily cross the BBB affecting the hypothalamic region by inducing intracellular acidification and alteration of neurotransmitter releases. In addition, PA was chosen for this study because it has a high brain uptake index (43.53%). Despite neurotoxic effects associated with the oral administration of PA to rats were also reported, however, this unnecessary action strictly depends on the dosage and duration [20]. It was not surprising that metformin and PA treatment did not influence nor body weight, waist, and length of rats neither glucose and HbA1c level; however, a slight tendency to the reduction of glycosylated hemoglobin after combined PA and metformin administration was observed.

New observations suggest that chronic hyperglycemia disrupts normal ER functioning and unfolded protein response signaling [7, 32]. Persistent ER stress has emerged as a key mechanism that contributes to insulin resistance and is involved in the development of obesity and T2DM. First, to investigate at the cellular level possible changes in the VMH cytoarchitectonics and ER ultrastructure in the hypothalamus of rats with T2DM and the influence of the metformin and PA administration, we performed an examination of VMH by electronic microscopy and an assessment of morphometric parameters of ER.

We observed HFD-induced profound changes in ER: tubules and cisterns of ER in most neurons were enlarged; an accumulation of fragmented mitochondria with signs of cristae *destruction* and matrix swelling was detected; nuclei were pyknotically altered, and deep invaginations of the nuclear membrane were detected. In the T2DM group, most neurons had signs of apoptosis and dendritic loss. Moreover, we found morphopathological changes of glial cells in VMH of rats after HFD—swelling of the cytoplasm and a reduced number of organelles. This is in line with recent data allowing to consider diabetes as a disease of ER stress; however, it is mostly confirmed for *β*-cells [33, 34]. Moreover, earlier studies in peripheral tissues have established ER stress signaling as a key proinflammatory mediator in the pathogenesis of overnutrition-related diseases [35]. Particularly, pharmacologic or genetic induction of ER stress in the hypothalamus can mimic metabolic inflammation to cause central leptin and insulin resistance, resulting in a broad range of metabolic disorders including overeating, glucose intolerance, and hypertension [36].

However, despite the confirmation of the development of ER dysfunction after HFD, we also obtained completely new data concerning the influence of metformin and PA on the morphology of ER in the VMH. We found that the administration of drugs partially improved the morphological signs of damage in the hypothalamus by reducing mitochondrial swelling, decreasing the number of apoptotic neurons and also by enhancing the glial response. It is not surprising since earlier, it was shown that metformin demonstrated neuroprotective effects, however not *in vivo* but on primary cortical neurons [37]. However, to our knowledge, there are no studies related to ER morphology in VMH after metformin treatment on the background of T2DM. The hallmarks induced after metformin administration were an increase in the number of lysosomes and autophagosomes and their accumulation in axoplasm, swelling of myelinated *fibers*, and the reduction of the volume and relative area of the ER cisterns compared with the T2DM group. In addition, after metformin administration, there was an accumulation of lipofuscin in neurons, which is considered a sign of “cell aging,” and also may inhibit the ubiquitin-proteasomal system, adequate stress response, and increase oxidation of unsaturated fatty acids, which altogether lead to neuronal degeneration. Therefore, despite the effective reduction of hyperglycemia, metformin monotherapy does not prevent the development of diabetes-induced hypothalamic impairments. Another interesting observation after metformin treatment was the presence of reactive astrogliosis, characterized by an increase in the number of astrocytes and their hypertrophy. These visual observations were confirmed by a profound elevation of GFAP protein level, which is considered one of the markers for the activation of *astrocytes* following injury or stress.

Since it has been reported previously that *propionic acid* may reduce *ER* stress in bovine mammary epithelial cells [23], we examined the influence of PA on ER morphology in rat VMH and found that PA administration on the background of T2DM caused the similar effects on ER architectonics as it was observed in metformin group but in greater extent. The relative area of ER membranes and cisterns as a total fraction of ER in the cells was progressively decreased that was combined with an increase in the number of autophagosomes and a decrease in neuronal apoptosis after PA action compared with diabetes. Additionally, PA administration led to a decrease in the number of apoptotic dark neurons with pyknotically altered nuclei compared with diabetes. The point that needed to be emphasized here is that the difference compared with metformin was that astrocytic glia demonstrated no signs of swelling. Based on these observations and our previously published data as conference abstract [38], it can be assumed that PA activates antiapoptotic mechanisms in neurons by stimulating the switch to autophagy as an important protective way to prevent an accumulation of misfolded proteins. These statements require further research to understand the regulation of the tight balance between apoptosis and autophagy in VMH under the influence of PA.

Based on the electron microscopy data, showing the effects of separate metformin and PA administration after HFD-induced diabetes, we expected that their combination would be more effective. Nevertheless, after combined treatment, we found an increase in the quantity of microglial cells, preapoptotic, and apoptotic neurons that in turn may activate microglia. Interestingly, it should be noted the profound lowering effect of combined administration of PA+metformin on the relative area of ER membranes and cisterns compared with all 3 groups (control, T2DM, and metformin). Thus, an interesting new trend was observed under the influence of compounds: after T2DM-induced elevation, three ER parameters (relative area of ER membranes and cisterns and the total fraction of ER in the cell) were lowered to a different extent after the treatment with metformin, PA, and their combined exposure. When ER homeostasis is disrupted, the ER activates adaptive signaling pathways, called the unfolded protein response. Taken together, these observations allowed us to assume that PA and metformin may also influence not only ER stress and ER parameters on the cellular level but also affect the adaptive UPR, which play a crucial role in protein folding and maintenance of cellular homeostasis.

Chronic ER stress and defects in UPR signaling are currently considered key contributors to a growing list of human diseases, including neurodegeneration, cancer, and diabetes. Recent evidence showed that ER stress and abnormal UPR activation in the hypothalamus are involved in central leptin/insulin resistance in obesity and T2DM [39]. Administration of HFD to rats rapidly triggers inflammatory signaling in the hypothalamus [40], and hypothalamic inflammation was detected in the brains of obese humans, further implicating hypothalamic dysfunction in the pathogenesis of obesity [41]. Moreover, it was shown that transient hypothalamic ER stress may be a trigger to induce peripheral glucose intolerance and increased plasma levels of noradrenaline in mice [42]. To date, much interest is dedicated to targeting UPR components as a therapeutic strategy to combat ER stress-associated pathologies [43]. Since we observed that the administration of metformin and PA caused multidirectional changes in the ER parameters, our next step was to assess UPR-related gene and protein expression following metformin and PA treatment in VMH of rats.

Our observations revealed an interesting trend showing that changes in the mRNA levels of the UPR system components have appeared insignificantly in the maximum of 36-38% from control; thus, mRNA pool of UPR components was stable; however, at the protein level, the difference was up to 5-fold. GRP78 is a master regulator for ER stress and a major ER chaperone with antiapoptotic properties and the ability to control the activation of UPR [44]. When protein folding occurs normally in the ER lumen, GRP78 is bound to a triad of stress-sensing proteins—PERK, IRE1, and ATF6, and keeps them in inactive states. Upon ER stress, GRP78 has released from ER transmembrane signal transducers and led to an activation of its downstream cascades; thus, the level of GRP78 is used as an indicator of ER stress [45]. Unexpectedly, we found that the protein level of GRP78 in VMH of T2DM rats was significantly lower than in the control. In contrast, in other studies on STZ-treated diabetic rats, UPR-related proteins, such as GRP78/BiP, were increased in glomerular and tubular cells [46] and BiP, CHOP, p-PERK, and p-eIF2*α* levels were increased in the diabetic mice [47], demonstrating diabetes-related augmentation of ER stress. We suggested that the observed decrease in GRP78 level might be explained by its active binding to accumulated misfolded proteins to target them to ER-associated protein degradation, and thus, GRP78-mediated downstream signaling pathways should be overactivated.

The next part of our data is in line with the abovementioned works, and we definitely observed that levels of other UPR components were increased, especially those that regulate the expression of GRP78—ATF6 і IRE1, thus confirming the activation of UPR and proteotoxic ER stress under T2DM. Interestingly, despite that ATF6, which upregulates proteins like ER chaperones including GRP78, was increased, the level of GRP78 was still lower than in control. Possibly, it may reflect UPR time-dependent response and/or the block of GRP78 protein synthesis, since *Grp78* mRNA was unchanged in the T2DM group. Diabetes-induced elevation of ATF6 level correlated with an enlargement of ER volume and a dilatation of cisterns that reflects the expansion of functional ER capacity. It is also unquestioned that ATF6 activity influences the cell fate between survival and death, and regarding the data from electron microscopy, there is a tendency to cell death. Another interesting point was an excessive increase in the level of PERK, which prevents further protein synthesis and may represent an adaptive response. However, it may also reflect nonadaptive, but harmful effect: when PERK activity is chronic, sustained ATF4 levels upregulate proapoptotic proteins such as CHOP and growth arrest and DNA damage-inducible 34 (GADD34) and this may reflect cocalled “PERK-opathy” state in which PERK mediates detrimental effects leading to chronic disorders [48, 49]. Since the IRE1 level was also significantly increased in diabetic rats, its overexpression may act as a potent inducer of cell death [50] that was also in line with our data from electron microscopy examination of VMH.

Persistent UPR activation plays a paradoxical role and triggers cell death signaling pathways, apoptosis, and concomitant tissue injuries [51]. Therefore, it was an important question to address whether metformin and PA or their combined treatment may prevent overactivation of UPR signaling and restore cell homeostasis. Interestingly, there are several works related to the diminishing effect of metformin on ER stress in addition to its well-known antidiabetic effect. Thus, metformin can decrease ER stress in renal cells [52], angiotensin II-induced ER stress, and hypertension in mice through activation of AMPK [53] and also decrease *β*-cell lipotoxicity through inhibition of the ER stress [54]. In this study [55], chronic feeding with metformin decreased the ER stress-induced apoptosis in mouse hearts, indicating that targeting the ER stress is a potential approach to decrease the occurrence or progression of heart failure.

In line with these data, in our study, we showed that metformin administration restored GRP78 protein level to control value, leading to a decrease in ATF6 and IRE1 contents compared with diabetes; however, PERK level was further increased. We hypothesized that PERK signaling in this condition may play a protective role because it reduces the synthesis of misfolded proteins. This hypothesis is also supported by the study showing that the PERK-eIF2*α*-ATF4 pathway induced by ER stress may alleviate chronic renal failure-induced hippocampal neuronal damage [56]. Thus, metformin acts on the GRP78 level, and despite a partial decrease in ATF6 and IRE1, a UPR overactivation still was present. What is interesting here is the further elevation of PERK level compared with the T2DM and control groups. This is in agreement with the study, in which authors demonstrated that metformin activates the PERK-ATF4 but not the ATF6 or IRE1-XBP1 branches and leads to a strong upregulation of CHOP mRNA and protein in primary rat cardiomyocytes [57].

Interestingly, PA administration upon T2DM further increased GRP78 and PERK levels; however, a profound lowering effect of PA on IRE1 and especially on ATF6 (even lower than in control) was detected. Thus, PA mostly acts on the ATF6 branch and ATF6 signaling after treatment declined, yet PERK signaling persisted much longer in the presence of unmitigated ER stress. PERK may facilitate ATF6 function by enhancing the transport of ATF6 from the ER and Golgi [58]. Further elevation of GRP78 restored its potential to bind three UPR sensors and thus lowering its overactivation under T2DM condition.

The most important issue was to assess the action of combined treatment with metformin and PA on the state of the hypothalamic UPR system. Metformin and PA combination restored ATF6 and IRE1 to control values and lowered PERK level more intensively, than when administered separately. Moreover, combined administration led to an elevation of GRP78 level even compared with control, suggesting more effective binding to three UPR signaling sensors, keeping them in an inactive state, inhibiting UPR overactivation, more effective binding to abnormally folded proteins. Therefore, we can speculate that these effects may altogether mediate a neuroprotective action. These data are in agreement with this study [59], which showed that region-specific alleviation of ER stress in the hypothalamus was due to GRP78 overexpression and was accompanied by reversion of the obese and metabolic phenotype in mice, thus confirming that GRP78 overexpression may exert a protective effect. Moreover, here [60], the authors demonstrated that GRP78 overexpression reduces ER stress markers in the liver of obese mice. In addition, hypothalamic ER stress suppression could represent a potential strategy to combat obesity, MS, and other diseases [61]. Therefore, we can suggest that inhibiting UPR activation by the GRP78 overexpression may have beneficial effects on ER stress in VMH induced by HFD. To conclude, the action of drugs on the UPR signaling was as follows: metformin and PA had the lowering effect on ATF6 and IRE1 in different extent, however increased PERK and GRP78. Combined PA and metformin action was more effective than a separate one; however, the mechanisms through which they act still required further elucidation. Taken together, these data suggest that impairment of hypothalamic UPR under chronic HFD feeding was induced mainly through posttranslational processes rather than gene transcriptional processes, and the influence of drugs has mainly appeared on the protein level.

An enormous volume of literature is dedicated to the elucidation of molecular mechanisms of hypothalamic insulin resistance [62] including hypothalamic gliosis. We studied the effects of PA and metformin on astrocyte/microglia crosstalk in the VMH of rats with T2DM. To our knowledge, there have been no reports describing whether PA has an influence on glial cells. First, we determined the level of the microglial marker Iba1. The Iba1 protein expression pattern was similar to PERK—an elevation in the T2DM group with further increase after metformin and PA action (even on the mRNA level), suggesting an overactivation of microglia. We can suggest that HFD-induced microglial activation in the hypothalamus may be a manifestation of neuronal damage, which in turn triggers reactive gliosis involving both microglial and astroglial cell populations. By contrast, combined treatment exerted a lowering effect on the Iba1 level, which was confirmed immunohistochemically; however, it was still higher than in the control group.

Interestingly, there was a study showed that HFD feeding for one day is enough to induce hypothalamic gliosis, including both microgliosis and astrogliosis [40, 41]. Microgliosis is induced by HFD feeding, but not obesity [63], and contributes to hypothalamic inflammation [64, 65]. In contrast, it has been demonstrated that astrogliosis is recognized as a protective reaction of the brain responding to acute excess nutrition [66]. We assessed the level of GFAP, a main intermediate filament protein in mature astrocytes, and found that its content did not change in the T2DM group, while separate drug administration led to an increase in GFAP level in VMH. GFAP elevation was more pronounced after PA, suggesting its protective effect, that was also in agreement with our data that astrocytic glia after PA treatment demonstrated no signs of swelling compared with the metformin group. Combination of PA and metformin was not so effective as a separate PA action; however, GFAP content was still 1.78-fold higher than in control. Observed microglial activation may point to the development of the inflammatory process in the hypothalamus. However, it was shown that inflammation and further activation of nuclear factor *κ*B- (NF-*κ*B-) associated pathways in astrocytes plays a controversial role, because from the one side, one report has shown that the inhibition of NF-*κ*B in astrocytes enhanced food intake [67], and from the other, this study demonstrated astrocytic-mediated inhibition of NF-*κ*B to protect animals from HFD-induced obesity [68, 69]. Thus, based on our observations from electron microscopy, we suggest that on the background of microglial activation, observed drug-induced astrogliosis may reflect a protective response to T2DM-associated neurological injury and contribute to the restoration of VMH homeostasis; however, there is no full restoration of morphopathological changes after HFD-induced hypothalamic impairments.

An additional pathological mechanism that may be involved in the diabetic-induced hypothalamus impairments is the disturbance of cell integrity. ZO-1 is a protein involved in the assembly and proper function of a number of tight junctions and is also expressed in astrocytes and microglial cells [29, 70]. There is interesting data that diabetes and high glucose concentration may induce a decrease in ZO-1 in rat glomerular epithelial cells of animal models of both type 1 and 2 diabetes [71]. Moreover, another study has shown that an alteration of ZO-1 may participate in the pathogenesis of BBB dysfunction, cerebrovascular damage, and diabetes-associated stroke [72]. Our data was in line with these observations, and we found a T2DM-induced decrease in ZO-1 level that correlated with microglial activation. Interestingly, under systemic inflammation, microglia are able to phagocytose the *astrocytic endfeet*, which leads to a violation of the density of cell connections and impairs BBB functioning [73]. Metformin did not affect ZO-1 level compared with diabetes, while PA treatment exerted a significant increasing effect. These data are in agreement with the study on rats, in which authors showed that continuous oral administration of PA during lactation may increase its concentration in the proximal colon and promote epithelial barrier function of proximal colon by enhancing the expression of ZO-1, claudin-8, claudin-1, and occludin [74]. Expectedly, after combined treatment of diabetic rats, the effects of metformin and PA were summarized, and the ZO-1 content was higher than in the metformin group but lower than in the PA group. Thus, our findings showed T2DM-induced activation of microglia and impairments in cell integrity of VMH. The combination of PA and metformin effectively lowered the Iba1 levels and partially restored ZO-1content, suggesting their perspective immunomodulatory and neuroprotective effects.

It should be noted, that due to technical limitations, such as the small weight of rat VMH, we cannot perform the RT-PCR of all markers, detected by western blotting. It also remains partially elucidated by which mechanism PA exerts its effects on VMH; therefore, additional investigations are required.

## 5. Conclusions

T2DM-induced imbalance of UPR signaling, activation of microglia, and impairments in cell integrity may trigger nerve cell dysfunction in VMH. Diabetes-induced imbalance between the components of the UPR system in VMH was more pronounced at the protein level than at the transcriptional, indicating a significance of UPR homeostasis. Drug administration slightly improved ultrastructural changes in VMH. Treatment with metformin and PA partially normalized the ratio between UPR sensors and regulators; however, it still remains unclear which signaling pathway was more involved in the compensatory response. Nevertheless, PA and its combination with metformin may have beneficial effects for counteracting diabetes-induced ER stress and normalizing astrocyte/microglia crosstalk in VMH.

## Figures and Tables

**Table 1:** Primers used in this study.

Primer name	Forward primer sequence (5′→3′)	Reverse primer sequence (5′→3′)
*Grp78*	TCGACTTGGGGACCACCTATTCC	GCCCTGATCGTTGGCTATGATCTC
*Perk*	CAGAGAAGTGGCAAGAGGAGATGGA	GGGCATCCATTGGGCTAGGG
*Atf6*	AGCTGGACCAGGTGGTGTCAGAG	CACAGACAGCTCTGCGCTTTGG
*β-Actin*	TGCAGAAGGAGATTACTGCCCTGG	GCTGATCCACATCTGCTGGAAGG

**Table 2:** The animal body parameters, blood glucose, and HbA1c after diabetes induction.

	Control group	T2DM group	T2DM+metformin group	T2DM+PA group	T2DM+metformin and PA group
Weight, g	176.8 ± 8.38	291.2 ± 17.63^∗^	294.0 ± 8.43^∗^	288.6 ± 9.82^∗^	267.2 ± 25.41^∗^
Length, cm	20.0 ± 0.31	23.0 ± 0.63^∗^	21.6 ± 0.98^∗^	22.83 ± 0.85^∗^	22.4 ± 0.94^∗^
Waist, cm	11.8 ± 0.48	16.0 ± 0.31^∗^	15.5 ± 0.22^∗^	16.08 ± 0.45^∗^	15.1 ± 0.81^∗^
Blood glucose level, mmol/l	4.83 ± 0.39	9.55 ± 0.59^∗^	9.46 ± 1.34^∗^	10.5 ± 0.55^∗^	9.06 ± 0.69^∗^^,@^
Blood HbA1c level (%)	5.17 ± 0.69	9.01 ± 0.85^∗^	8.8 ± 1.16^∗^	10.9 ± 0.87^∗,∗∗^^,#^	7.98 ± 0.42^∗,∗∗^^,@^

Note: Values are given as mean ± SEM (*n* = 6); ^∗^*p* < 0.05 vs. control, ^∗∗^*p* < 0.05 vs. T2DM, ^#^*p* < 0.05 vs. metformin administration, and ^@^*p* < 0.05 vs. PA administration.

**Table 3:** Structural ER parameters after metformin and propionate administration on the background of T2DM.

Groups	Control group	T2DM group	T2DM+metformin group	T2DM+PA group	T2DM+metformin and PA group
Relative area of ER membranes (S_membr_)	0.453 ± 0.05	0.488 ± 0.02	0.379 ± 0.06^∗∗^	0.327 ± 0.05^∗,∗∗^^,#^	0.302 ± 0.03^∗,∗∗^
Relative area of ER cisterns (S_cist_)	0.052 ± 0.01	0.117 ± 0.02	0.086 ± 0.01^∗,∗∗^	0.037 ± 0.006^∗,∗∗^^,#^	0.034 ± 0.008^∗,∗∗^^,#^
Ratio S_cist_/S_membr_	0.114 ± 0.028	0.239 ± 0.053	0.226 ± 0.019^∗^	0.113 ± 0.006^∗,∗∗^^,#^	0.112 ± 0.019^∗,∗∗^
Total fraction of ER in the cell	0.505 ± 0.059	0.605 ± 0.038	0.465 ± 0.076	0.364 ± 0.063^∗,∗∗^	0.336 ± 0.045^∗,∗∗^
Ratio of the area of the perinuclear space/length of the nucleus perimeter	0.034 ± 0.005	0.049 ± 0.012	0.038 ± 0.009	0.019 ± 0.005^∗,∗∗^^,#^	0.027 ± 0.007^∗^

Note: Values are given as mean ± SEM (*n* = 12); ^∗^*p* < 0.05 vs. control, ^∗∗^*p* < 0.05 vs. T2DM, ^#^*p* < 0.05 vs. metformin administration, and ^@^*p* < 0.05 vs. PA administration.

## Data Availability

The data supporting the findings of the study readers can access from the corresponding author by personal request.
